# Interactions Between Glycogen Synthase Kinase-3β Gene Polymorphisms, Negative Life Events, and Susceptibility to Major Depressive Disorder in a Chinese Population

**DOI:** 10.3389/fpsyt.2020.503477

**Published:** 2021-02-15

**Authors:** Jiarun Yang, Siyuan Ke, Zhengxue Qiao, Xiuxian Yang, Xiaohui Qiu, Xuejia Song, Erying Zhao, Jiawei Zhou, Mingzhe Zhao, Yanjie Yang, Deyu Fang, Depin Cao

**Affiliations:** ^1^Department of Health Management, Public Health Institute, Harbin Medical University, Harbin, China; ^2^Department of Psychology, Public Health Institute, Harbin Medical University, Harbin, China; ^3^Northwestern University Feinberg School of Medicine, Evanston, IL, United States

**Keywords:** glycogen synthase kinase-3β, polymorphism, major depressive disorder, gene-environment interaction, negative life events

## Abstract

**Background:** Recent studies suggest that glycogen synthase kinase (GSK)-3β is involved in the development of major depressive disorder (MDD). The aim of this study was to investigate the interaction between GSK-3β polymorphism (rs6438552, rs334558, and rs2199503) and negative life events in the pathogenesis of major depressive disorder (MDD).

**Methods:** DNA genotyping was performed on peripheral blood leukocytes in 550 patients with MDD and 552 age- and gender-matched controls. The frequency and severity of negative life events were assessed by the Life Events Scale (LES). A chi-square method was employed to assess the gene-environment interaction (G × E).

**Results:** Differences in rs6438552, rs334558, and rs2199503 genotype distributions were observed between MDD patients and controls. Significant G × E interactions between allelic variation of rs6438552, rs334558, and rs2199503 and negative life events were observed. Individuals with negative life events and carrying genotypes of rs6438552 A^+^, rs334558 A^+^, and rs2199503G^+^ have increased the risk of depression.

**Conclusions:** These results indicate that interactions between the GSK-3β rs6438552, rs334558, and rs2199503 polymorphisms and environment increases the risk of developing MDD.

## Introduction

Major depressive disorder (MDD) is a group of mental disorders characterized by persistent low emotions, often accompanied by slow thinking, delayed behavior, and various somatization symptoms (1). MDD has a high prevalence, high recurrence rate, and high suicide disability rate, but there is a low detection rate, low treatment rate and low cure rate. The lifetime prevalence of MDD is 12–17% (2). Although the etiology of MDD remains unclear, a genetic component is likely to contribute to the development of MDD, with heritability ranging from 40 to 50% (3, 4). Thus, the identification of MDD susceptibility genes not only makes it easier to explore the etiology of depression, but also can better reduce the prevalence of depression and provide targeted treatment to patients.

As people continue to explore the hypothesis related to depression, namely the hypothesis of mental illness and neuroplasticity, related research on glycogen synthase kinase (GSK-3β) (a key regulator of neuronal function) has increased (5). Studies have shown that GSK-3β may be related to the pathogenesis of MDD. Previous studies have shown that GSK-3β may be environmentally sensitive. Studies have reported that the combined effects of BDNF and GSK-3β genes regulate the relationship between negative life events and MDD (6). In addition, the latest research also shows that inhibiting the activity of GSK-3β may affect the therapeutic effect of antidepressants. Treatment with antidepressants inhibits GSK-3β activity in the mouse brain (7, 8). In conclusion, GSK-3β may be a susceptibility gene for depression and is involved in the pathophysiological and therapeutic processes of MDD.

The GSK-3β gene is located on the chromosomal region, 3q13.3, and is widely expressed in all brain regions. After rapid anti-depression treatment with ketamine, the phosphorylation level of GSK-3β in plasma increases significantly, suggesting that GSK-3β may be involved in the mechanism underlying anti-depression (9). GSK-3β gene single nucleotide polymorphisms [SNPs] (rs6438552, rs2199503, and rs334558) are associated with symptoms of depression in the Chinese population (10). The rs6438552 allele is closely related to the superior temporal gyrus of patients with depression and the decline of gray brain volume in the right hippocampus, and it was suggested that GSK-3β could be a susceptibility gene to MDD in the Chinese Han population (11). Herein, we studied the association between GSK-3β SNPs with MDD and investigated the effects of common confounding factors in conjunction with the SNPs on the susceptibility to MDD.

Although 31–42% of MDD cases are affected by genetic susceptibility, external environmental factors also play a vital role in the pathogenesis of MDD (12). More and more studies have shown that stress is an important factor in inducing the onset of MDD (13, 14). It induces negative emotional experiences such as sadness, loneliness, anxiety, depression, etc. by affecting the cognitive evaluation system. This leads to a series of physiological, biochemical, and immune system changes. Studies have shown that patients with MDD experience more negative life events than normal people. Therefore, negative life events are important triggers of MDD. The more negative life events, the more serious the nature, the higher the incidence of MDD, and the more severe the symptoms of MDD. There is a relationship between quantity and response.

Therefore, the interaction between environmental and genetic factors is the key reason for the pathogenesis of MDD. The purpose of this study was to investigate the interaction between GSK-3 antidepressants and negative life events in Chinese population, and to determine whether this interaction is involved in the pathophysiological process of MDD.

## Materials and Methods

### Subjects

The study included 550 patients with MDD and 552 healthy controls. Both the patients and the control group were han residents in northern China.

The MDD group consisted of 165 males and 385 females [mean age, 44.53 years (S.D., 13.53); age range, 16–30 years]. All patients with depression had their first onset and underwent a structured interview by two experienced psychiatrists using the Diagnostic and Statistical Manual of Mental Disorders (DSM-V) to confirm MDD (15). The severity of depression was evaluated by a Chinese version of the 24-item Hamilton Rating Scale of Depression (HRSD-24). Patients with a minimum score of 21 were included in the study. Patients with other co-morbid axis-I disorders, a family history of genetic disease, neurologic diseases, and those who received an antidepressant medication within 4 weeks were excluded from the study.

The control group included 195 males and 357 females [mean age, 43.19 years (S.D., 9.08); age range, 16–30 years]. All subjects were recruited from the Physical Examination Center in the First Affiliated Hospital of Harbin Medical University. The Structured Clinical Interview for DSM-V (SCID) was used to exclude psychiatric diseases, neurologic illnesses, and alcohol or drug abuse. Controls were matched for age, gender, and level of education with the MDD patients. The study was approved by the Ethics Committee of the Harbin Medical University and all subjects gave written consent.

### Assessment of Negative Life Events

The Life Events Scale (LES) was used to assess negative life events (16), which has been verified in Chinese population. LES questionnaire has a total of 48 questions, involving the following three factors: family life (28 questions); Work (12); Social and other aspects (7 items). Negative life events include bereavement, divorce, serious illness, housing and financial crises, unemployment, work stress and problems with co-workers. Their scores were assessed by the interviewers themselves, based on how they felt, to produce a total life events score. This scale evaluates life events in the following four aspects: time of occurrence (absence = 1; So >1 year ago is equal to 2; In the past year =3; And chronic = 4); Character (good = 1; Bad = 2); Effect on mood (absence =1; Mild = 2; Gentle = 3; Serious = 4; And extreme = 5); Duration of influence (≤3 months =1; 3–6 months = 2; 6–12 months = 3; >12 months =4). The 75% percentile (2 points) was used as a threshold for high or low negative life events.

### DNA Extraction and Genotyping

Genomic DNA was extracted from collected venous blood samples using the AxyPrep Blood Genomic DNA Miniprep Kit (Axygen, Union City, CA, USA) and the SNPs (rs6438552, rs2199503, and rs334558) of the GSK-3β gene were selected for subsequent studies. The primers used for PCR amplification were designed using Primer 5.0 software, and the specificity of a potential primer was checked using the Basic Local Alignment Search Tool (BLAST) provided by the National Center for Biotechnology Information. The primers for amplifying the GSK-3β rs6438552 fragments were 5′-TCTAAACCTTAAAGAACTTAG-3′ (forward) and 5′- TCTTTTTTGCAGAGCAAGGTG-3′ (reverse). The primers for amplifying the GSK-3β rs334558 fragments were 5′-TCAGGAAGTGTCCGCGCTTTG−3′ (forward) and 5′-AAGGAGGTGGAGGACGAGTAG−3′ (reverse). The primers for amplifying the GSK-3β rs2199503 fragments were 5′-TCAGGAAGTGTCCGCGCTTTG−3′ (forward) and 5′-AAGGAGGTGGAGGACGAGTAG−3′ (reverse). Analyses of SNPs was performed using SNaPshot according to the manufacturer's instructions.

### Statistical Analysis

The SPSS package (version 20.0 for Windows) was used for data analyses. A chi-square (χ^2^) goodness-of-fit test was performed to test the Hardy-Weinberg equilibrium (HWE) for the genotypic distribution of SNP. The χ^2^-test was used to compare SNP genotype and allele frequencies between MDD patients and controls. The generalized multi-factor dimension reduction (GMDR) was performed to analyze gene × environment interactions. All tests were two-sided, and statistical significance was set at a *P* < 0.05.

## Results

### Comparison of Demographic Data and Negative Life Events Between the Case and Control Groups

There was no significant difference between MDD patients and control subjects with respect to gender, age, and level of education (*P* > 0.05; Table 1); however, there were differences in negative life events between the two groups (*P* < 0.05; Table 2).

**Table 1:** Demographic data of MDD and control groups.

	**MDD (*n* = 550)**	**Control (*n* = 552)**	**χ^**2**^**	***p***
Sex			3.553	0.059
Male	165 (30.00%)	195 (35.30%)		
Female	385 (70.00%)	357 (64.70%)		
Age			0.902	0.637
16–30	111 (20.18%)	99 (17.93%)		
31–54	277 (50.36%)	286 (51.81%)		
>54	162 (29.46%)	167 (30.36%)		
Education			3.317	0.190
Junior school and below	288 (52.36%)	271 (49.09%)		
Senior school and Junior college	191 (34.73%)	220 (39.86%)		
College and above	71 (12.91%)	61 (11.05%)		

**Table 2:** Comparison of negative life events between the case and control groups.

**Negative life events**	**MDD (*n* = 550)**	**Control (*n* = 552)**	**χ^**2**^**	***p***
0	350 (63.6%)	414 (75.0%)	16.730	0.000
1	200 (36.4%)	138 (25.0%)		

### Effect of GSK-3β rs6438552, rs334558, and rs2199503 on MDD Susceptibility

The genotypic distributions of the GSK-3β polymorphisms conformed to the HWE for controls. Comparison of genotypes and alleles of the GSK-3β rs6438552, rs334558, and rs2199503 gene polymorphisms between the depression and control groups are summarized in Table 3. The genotype frequency distribution differences of the GSK-3β rs6438552, rs334558, and rs2199503 gene polymorphisms between the case and control groups were statistically significant (*P* < 0.05). The allele frequency distribution of the GSK-3β rs6438552, rs334558, and rs2199503 gene polymorphisms between the case and control groups was statistically significant (*P* < 0.05).

**Table 3:** Comparison of genotypes and alleles of GSK-3β rs6438552, rs334558, and rs2199503 gene polymorphisms between the depression and control groups.

**SNPs**	**Group**		**Genotype frequencies**		***p***	**Allele frequencies**		***p***
rs6438552		AA	AG	GG		A	G	
	MDD	132 (24.0)	316 (57.5)	102 (18.5)	0.00	580 (52.7)	520 (47.3)	0.00
	Control	98 (17.8)	245 (44.3)	209 (37.9)		441 (39.9)	663 (60.1)	
rs334558		AA	AG	GG		A	G	
	MDD	109 (19.8)	300 (54.6)	141 (25.6)	0.00	518 (47.1)	582 ((52.9)	0.00
	Control	81 (14.7)	243 (44.0)	228 (41.3)		405 (36.7)	699 (63.3)	
rs2199503		GG	AG	AA		G	A	
	MDD	97 (17.6)	293 (53.3)	160 (29.1)	0.00	487 (44.3)	613 (55.7)	0.00
	Control	68 (12.3)	233 (42.2)	251 (45.5)		369 (33.4)	735 (66.6)	

### Combined Effects of GSK3β rs6438552, rs334558, and rs2199503 Polymorphisms and Negative Life Events on MDD

According to the dominant genetic model, the three SNPs of the GSK-3β gene are divided into two types {primitive type and mutant type [rs6438552 A^−^(GG), rs6438552 A^+^(AG+AA); rs334558 A^−^(GG), rs334558 A^+^(AG+AA); rs2199503 G^−^(AA), and rs2199503 G^+^(AG+GG)]}. GMDR was used to analyze the interaction between the GSK3β rs6438552, rs334558, and rs2199503 gene polymorphisms with negative life events and depression, and the results showed that there are interactions between GSK-3β rs6438552, rs334558, and rs2199503 polymorphisms and negative life events. Individuals with negative life events and rs6438552 A^+^, rs334558 A^+^, and rs2199503G^+^ genotypes are at increased risk for depression (*P* < 0.05; Table 4).

**Table 4:** GMDR analysis of gene-environmental interactions in MD patients and controls.

**No. of factors considered**	**Best combination**	**Prediction error**	**Cross-validation consistency**	***P*-value**
3	rs334558A^+^, rs6438552A^+^, negative life events	0.3626	9/10	0.001
4	rs334558A^+^, rs6438552A^+^, rs2199503G^+^, negative life events	0.3672	10/10	0.001

## Discussion

Several lines of evidence indicate that GSK-3β is a good candidate for MDD susceptibility. In this study we determined whether or not common SNPs in the GSK-3β gene are associated with MDD and whether or not these three sites have interactions with negative life events. The results showed that there were interactions between negative life events and GSK-3β rs6438552, rs334558, and rs2199503 polymorphisms. Thus, individuals suffering from negative life events and carrying rs6438552 A^+^, rs334558 A^+^, and rs2199503G^+^ genotypes are at increased risk for depression.

Our results suggest that GSK-3β rs6438552, rs334558, and rs2199503 polymorphisms are a susceptibility site for depression. Karege et al. (17) have shown that the activity of GSK-3β is associated with depression. The hyperactivity of GSK-3β expressed in neurons causes cognitive impairment, tau hyperphosphorylation, increased β-amyloid production, neuronal death, and neuroinflammation leading to the occurrence of depression (18). In a study of GSK-3β polymorphisms, rs6438552, rs2199503, and rs334558 are considered to be involved in the treatment of patients with bipolar disorder (19). There is ample evidence that there is an overlap between depression and bipolar disorder, and genetic epidemiology studies, genomic linkage analysis, and genome-wide association studies (GWAS) support overlapping genetic factors between the two diseases. Inkster et al. (20) found that changes in the volume of gray matter (GM) in the right hippocampus and bilateral superior temporal gyrus (STG) in MDD patients were correlated with common intron polymorphism of GSK-3β (rs6438552). In a study of unipolar depressive patients, Tsai et al. (21) reported that structural abnormalities on MRI may be associated with the response to serotonin-reuptake inhibitors at 4 weeks and 3 SNPS in GSK-3β gene regions (15 SNPS detected). The study also found that rs6438552 was associated with gray matter volume in the right hippocampus and bilateral superior temporal gyrus (20). Taken together, these findings suggest that SNP rs6438552 of GSK-3β may transmit the risk of MDD by destroying neural connections. This finding is consistent with our findings. At the same time, we also found that rs2199503 and rs334558 had associations with MDD. A study by Jin et al. (22) in the Chinese Han population showed that rs2199503 is closely related to depression. This finding is related to the neurotrophic factor hypothesis for depression. Of note, the association between the rs2199503 single nucleotide polymorphism and the onset of depression through a large-sample case-control study [1,045 cases of depression vs. 1,235 cases of healthy controls; (22)], which is consistent with our results. Inkster et al. (10), however, did not find a correlation between rs2199503 SNP and gray matter volume in a study on the correlation between the GSK3β gene polymorphism and brain structure in patients with depression. For that reason, our study warrants replication in future studies. As a functional polymorphism within the GSK-3β promoter region, the GSK-3β rs334558 polymorphism might be a potential risk factor for MDD. A controlled study of 1,045 patients with MDD and 1,235 healthy volunteers found that rs334558 was associated with MDD in GSK3β patients (23). And this locus is a functional SNP that plays a role in the promoter. The A allele (primary allele) has previously been found to be associated with high GSK3β expression. Allele (A) of rs334558 may transmit the risk of MDD, and there was no significant heterogeneity in allele model and dominant gene model, indicating that rs334558 mutation was consistent with MDD association (24). In addition, the work of Levchenko et al. also confirms that the GSK3β gene polymorphism rs334558 may play a key role as a genetic and pharmacological biomarker in MDD (25). These conclusions provide the basis for our future research.

In addition, genetic factors have been shown to influence stress exposure (26). Genetic factors increase the risk of developing MDD by choosing high-risk environments. Although there have been a large number of studies on GSK-3β gene polymorphism in MDD patients, little attention has been paid to the interaction between GSK-3β gene polymorphism and negative sexual life events in these patients. Gene-environment interaction studies can explore how environmental factors influence depression. Our results showed that there was an interaction between rs6438552, rs334558, and rs2199503 polymorphism and negative life events in GSK-3β. This conclusion can be explained by diathesis-stress model (5). The model assumes that individuals with environmental susceptibility genes are more likely to develop MDD when they experience negative life events. Lin et al. (27) found that the interaction between GSK-3β rs2199503 and negative life events increased the risk of metabolic syndrome. A number of studies have shown that metabolic abnormalities occur in people with depression and the two diseases are mutually causal. Yang et al. (6) demonstrated a correlation between the interaction between GSK-3β rs2199503 and negative life events and the incidence of depression in a case-control study. These findings are all consistent with our findings.

This study has some limitations. First, the sample size is relatively small. Secondly, LES was filled in subjectively by the interviewees and collected questionnaires retrospectively. Therefore, there may be a bias in the recall, susceptible to subjective influence.

## Conclusion

In summary, the present study indicated that an interaction between GSK-3β rs6438552, rs334558, and rs2199503 polymorphisms and environmental factors increases the risk of MDD. Further studies are necessary to replicate our results in larger populations and other ethnic groups, as well as to elucidate the underlying mechanism of gene × environment interactions involved in MDD.

## Data Availability Statement

The datasets analyzed in this article are not publicly available. Requests to access the datasets should be directed to yanjie1965@163.com.

## Ethics Statement

The study was approved by the Ethics Committee of the Harbin Medical University and all subjects gave written consent. The patients/participants provided their written informed consent to participate in this study.

## Author Contributions

YY, DC, and DF conceived and designed the study, supervised the analysis, and interpreted the data. XY and XQ supervised the analysis, interpreted the data, and wrote the preliminary manuscript. ZQ, XS, and EZ supervised the data collection by JZ and MZ. SK and JY performed the test administration, compiled the data, and wrote the preliminary manuscript. All authors contributed to the writing and review of the manuscript and approved the final version.

## Conflict of Interest

The authors declare that the research was conducted in the absence of any commercial or financial relationships that could be construed as a potential conflict of interest.

## Abbreviations

GSK3β: Glycogen Synthase Kinase-3β
MDD: major depressive disorder
LES: Life Events Scale
BLAST: Basic Local Alignment Search Tool
HWE: Hardy-Weinberg equilibrium
GWAS: genome-wide association studies
SZ: schizophrenia
BD: bipolar disorder.
